# Isolation and Purification of Glucans from an Italian Cultivar of *Ziziphus jujuba* Mill. and In Vitro Effect on Skin Repair

**DOI:** 10.3390/molecules25040968

**Published:** 2020-02-21

**Authors:** Alessia Fazio, Chiara La Torre, Maria Cristina Caroleo, Paolino Caputo, Pierluigi Plastina, Erika Cione

**Affiliations:** 1 Department of Pharmacy, Health and Nutritional Sciences, Department of Excellence 2018–2022, University of Calabria, Edificio Polifunzionale, 87036 Rende (CS), Italy; latorre.chiara@libero.it (C.L.T.); mariacristinacaroleo@virgilio.it (M.C.C.); pierluigi.plastina@unical.it (P.P.); erika.cione@unical.it (E.C.); 2 Department of Chemistry and Chemical Technologies, University of Calabria, 87036 Rende (CS), Italy; paolino.caputo@unical.it

**Keywords:** *Ziziphus jujuba* Mill., glucans, scanning electron microscopy, keratinocytes, MTT assay, scratch assay, wound healing

## Abstract

Glucans possess a broad spectrum of biological activities. In this context, the present study was performed to isolate glucans from an Italian cultivar of *Ziziphus jujuba* Mill. at three different harvesting periods, in order to evaluate their effects on wound healing. The dry fruits were subjected to an alkaline extraction and then isolated glucans were purified by dialyzation. The crude and soluble samples were characterized by FT-IR and SEM analyses. Afterwards, total, α- and β-glucan content was measured using an enzymatic procedure. The results highlighted that the glucan amount increased as the maturation proceeded as well as the β-glucan percentage, which ranged from 48.2 at the first harvesting to 65.4 at the third harvesting. Furthermore, the effects of isolated glucans on the viability and migration of keratinocytes were evaluated using the in vitro MTT and scratch wound assays. The best proliferative effects on keratinocyte migration have been achieved with soluble glucans from third harvesting at 100 μM after 24 and 48 h (*** *P* < 0.001). The same treated group showed significant narrowing of the scratch area after 24 h and complete closure of the injury after 48 h. The findings highlighted the effectiveness of soluble glucans on regeneration of damaged skin.

## 1. Introduction

*Ziziphus jujuba* Mill. is one of the oldest medicinal plants with a long history of nutrition use as well as diseases treatment [1]. It grows typically in Asia, but also in Australia, the US, and Europe [2,3]. The health benefit of jujube fruits includes its anti-inflammatory [4], and antioxidant activities showing possible pharmacological application [5,6,7]. Jujube fruits is also applied to the promising area of dermocosmetics, which includes wound healing care [8]. This latter capability is due to several bioactive compounds present in the fruit [8,9]. In recent years, glucans of oats and barley, in particular those ones with a β conformation, have just been studied in wound healing, showing multifunctional activities [8,10,11]. Glucans are present in various plant-origin foods and therefore can potentially be extracted from a variety of food matrices [12]. Glucans are biologically active biopolymers that exist in different structural organizations, depending on the food matrices [13]. The biological effect of glucans is still stimulating large research efforts of the relationship of their structural characteristics and their ability to interact with biological receptors [14]. In particular, they activate the immune system and stimulate defensive response against wounding [8]. This effect is mediated by receptors also located on skin cells such as keratinocytes and fibroblasts [15]. The presence of wound stimulates the organism to develop healing mechanisms to regenerate impaired skin. The cellular events that occur imply any of three complex biological processes: Inflammation, proliferation, and remodeling [16]. After a wound, hemostasis and generation of inflammatory stimuli take place and then activated macrophages produce growth factors and cytokines, which stimulate anti-inflammatory and antibacterial effects, and the migration of dermal fibroblasts to the wound. In turn, differentiated dermal fibroblasts synthesize extracellular matrix, such as a collagen and elastin, and promote dermal contraction in order to accelerate wound closure [17]. β-Glucans have significant effects on human dermal fibroblast (HDF) proliferation, keratinocyte migration, and procollagen secretion [18]. On the other hand, glucans stimulate the production of growth factors essential for skin, promotes collagen biosynthesis, and ensure moisture and elasticity of skin [19]. For this reason, the present study was designed to perform isolation, purification, and characterization of glucans extracted from jujube fruits (*Ziziphus jujuba* Mill.) at three different harvesting period, and to evaluate their effect on skin repair using a human keratinocyte cell line. 

The further aim was also to verify if different percentages of alpha and beta forms, contained in the glucans extracted from jujubes, can influence the regeneration of damaged skin.

## 2. Materials and Methods

### 2.1. Chemicals and Reagents

Solvents of analytical grade for this study: Acetone, methanol, ethanol, demineralized water, hydrochloric acid, and dimethyl sulfoxide (DMSO) were purchased from Carlo Erba Reagent (Milan, Italy). Sodium carbonate, dialysis tube (Molecular Weight Cut Off 12400), and 3-(4,5-dimethylthiasol-2-yl)-2,4-diphenyltetrazolium bromide (MTT) were purchased from Sigma-Aldrich (Milan, Italy). The glucans content was determined by mushroom and yeast β-glucan kit (Cat. No. K-YBGL Megazyme International, Bray, County Wicklow, Ireland), based on the method published by McCleary and Codd [20]. Human immortalized HaCat keratinocyte cell line was obtained from CLS Cell Lines Service GmbH (Eppelheim, Germany) after a Material Transfer Agreement, and Dulbecco’s modified Eagle’s medium (DMEM) and fetal bovine serum (FBS) were obtained from Thermo Fisher Scientific (Waltham, MA, USA).

### 2.2. Plant Material

Ziziphus jujube fruits were harvested in the locality of Rombiolo (Latitude: 38°35′34″08 N; Longitude: 16°0′9″00 E, Calabria, Southern Italy), in the period from September to October 2017. The fruits were picked up from the same area three times, at a distance of 15 days from each other. The round-shaped drupes at first harvesting (J1) had green color; at second harvesting (J2) were yellow green with mahogany-colored spots; at the third harvesting (J3), the color was almost completely red. Twenty fruits were picked at random from a single tree in order to calculate the size: Fruit length and diameter were measured by using a vernier caliper (Table 1). The fruits were then washed and then preserved under nitrogen, frozen at −20 °C before undergoing the freeze drying process (Telstar freeze-dryer, mod. Cryodos, Terrassa, Barcelona, Spain). Furthermore, lyophilized fruits were pitted, manually ground in a mortar with a pestle, sieved through a 60-mesh screen, and stored at −20 °C as a flour.

### 2.3. Extraction of Glucans

**Figure 1:**
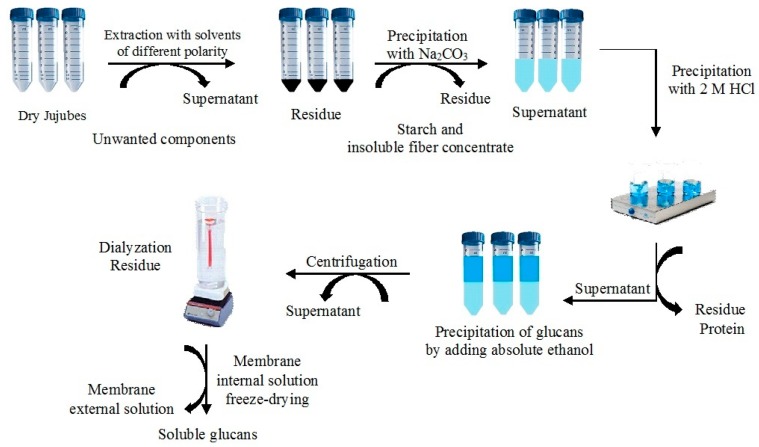
Scheme of the extraction of crude and soluble glucans.

The procedure used consisted of 3 phases: (a) Aqueous alkaline extraction of glucans and solubilized proteins, (b) acidic precipitation of proteins at their isoelectric point, and (c) precipitation of glucans by absolute ethanol [21]. The protocol for the sequential extraction of glucans from dried jujubes at laboratory scale is summarized in Figure 1. Briefly, J1, J2, and J3 flours (1 g) were subjected to purification phase in order to remove lipids, vitamin, polyphenols, monosaccharides, and disaccharides [22]. The phase consisted of extraction by using solvents at different polarity in sequence (acetone first, followed by methanol, and finally ethanol at 70% in bi-distillate water). The purification was carried out at room temperature (RT) for 2 h with each solvent (3 × 10 mL). The resulting solid residue was added with a solution of sodium carbonate (20% *w*/*v*) and then the mixture was stirred vigorously for 30 min in a water bath at 55 °C. The suspension was centrifuged for 30 min at 15,000 rpm (Model J2-21, Beckman Instrument Co., Mississauga, ON, Canada) and the supernatant pH was adjusted to 4.5 (isoelectric point of proteins) with 2 M of HCl. The mixture was re-centrifuged for 30 min at 15,000 rpm in order to separate the precipitated protein, which were thrown away [23]. The clarified supernatant, containing glucans fraction, was treated with an equal volume of ethanol 100% and left at 4 °C overnight. After that, the solution was centrifuged for 30 min at 15,000 rpm to separate the precipitate, containing crude glucans, which was washed twice with ethanol 100% and vacuum dried. The crude glucans were dialyzed, overnight at RT, by using a 12400 MCWO dialysis tubing, in order to remove low-molecular-weight molecules [24]. The solution from inside the dialysis tube was centrifuged for 30 min at 15,000 rpm and then the supernatant was freeze dried to calculate the yield of the water-soluble fraction. Both isolated fractions, crude and water-soluble glucans, were then characterized by FT-IR and SEM analysis. The extractions followed by purification were performed on three samples of jujube powder from each stage of harvesting.

### 2.4. Glucan Recovery Test

Recovery rate calculation was carried out in order to determine the validity and applicability of the experimental procedure. For this purpose, an experimental plan that involved the analysis of fortified samples, was realized. The fortified sample were prepared by spiking jujube flour at each stage of maturation (J1, J2, and J3) (1 g) at the beginning of the extraction procedure with different amount of glucan standard. Five spiking levels were performed: 30, 60, 90, 120 and 150 mg g^−1^. Four replicates for each spiking level were performed.

### 2.5. Detection of Glucan Content

**Figure 2:**
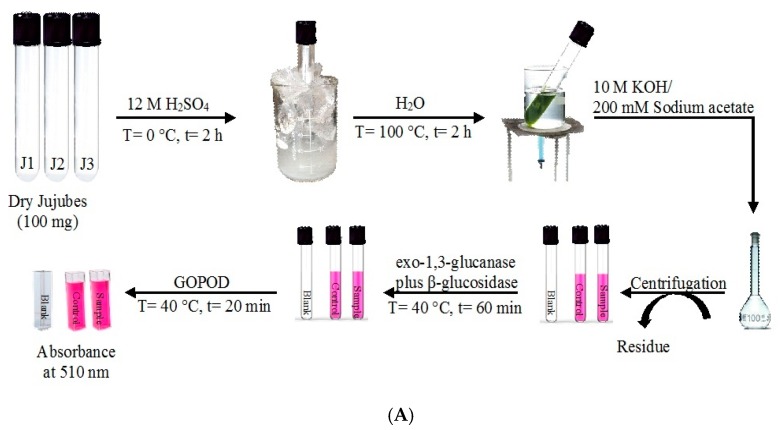

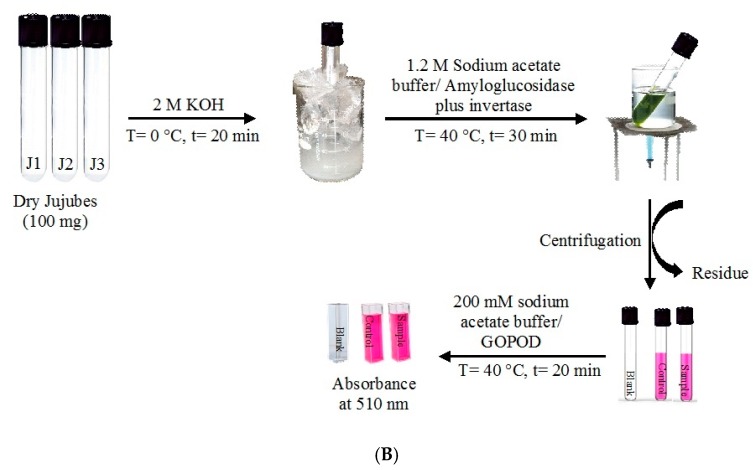
(**A**) Total glucan content and (**B**) α-glucan content.

The total glucan content was determined in triplicate using the β-glucan assay kit from Megazyme (Cat. No. K-YBGL, Astori Tecnica Snc, Poncarale (BS), Italy) [22]. The scheme of procedural sequences is shown in Figure 2A. Briefly, J1, J2, and J3 flours (0.1 g) were added with 12 M ice-cold sulphuric acid (2 mL). The mixture was stirred vigorously and incubated in an ice-cold water bath for 2 h. Then, distilled water (12 mL) was added to each sample and the suspension was kept at the boiling temperature in a water bath (T = 100 °C) for 2 h, shaking it occasionally in a vortex mixer during this time. After cooling to room temperature, each sample was added with 10M KOH (6 mL) and 200 mM sodium acetate buffer (pH 5.0) up to the volume of 100 mL. After centrifugation at 1500 rpm for 10 min, an aliquot of the supernatant (0.1 mL) was mixed with exo-1,3-β-glucanase (20 U/mL) plus β-glucosidase (4 U/mL) (0.050 ml) and incubated at 40 °C for 60 min. Then, the mixture was incubated at 40 °C for 20 min with glucose-oxidase/peroxidase-reagent (GOPOD, 3 mL). Total glucans were evaluated by the spectrophotometer analysis at λ = 510 nm (Model UV-vis, JASCO, V-550) against the blank (0.2 mL of 200 mM sodium acetate buffer at pH 5.0, plus 3 mL of GOPOD) and against the d-glucose standard solution (1 mg/mL), incubated with GOPOD reagent (Figure 2A). The α-glucan content was determined after incubation (T = 40 °C; t = 30 min) of the suspension of jujube flour (0.1 g) in 2M KOH (2 mL) added by 1.2 M sodium acetate buffer (pH 3.8, 8 mL) with 0.2 mL of a mixture of amyloglucosidase plus invertase. Each sample was centrifuged at 1500 rpm for 10 min and 0.1 mL of the supernatant were mixed with 0.1 mL of 200 mM of sodium acetate buffer (pH 5.0) and 3 mL of GOPOD (Figure 2B). β-Glucan content was determined by difference between total and α-glucan contents.

### 2.6. FT-IR Spectroscopic Analysis

Both crude and soluble glucans1 were characterized, according to their fingerprint, by FT-IR spectroscopy using a Bruker ALPHA FT-IR spectrometer (Billerica, MA, USA) equipped with a A241/D reflexion module. The sample and KBr were pulverized in an agate mortar and the powder was compressed with a press at a force of 6 tons [25]. Film spectra were recorded at 4000–400 cm^−1^ wavelength range and 4 cm^−1^ resolutions.

### 2.7. Scanning Electron Microscopy 

The characterization of sample surface morphology was acquired by a Scanning Electron Microscope (SEM) (Field Emission SEM FEI Quanta 200, Thermo Fisher Scientific, Hillsboro, OR, USA) and Electron Probe Micro Analyzer (EPMA, JEOL JXA 8230t, Kyoto, Japan), equipped with wavelength dispersive (WDS) in order to determine both the elemental composition and spatial distribution. The surface of the samples was coated by a 5 nm thick layer of Carbon, using a Carbon Coater QUORUM Q150T-ES (Darmstadt, Germany). The operating conditions for SEM analysis were the following: HV 15 KeV; Probe Current 10–20 nA; working Distance of 11 mm; image BSE signal; detector image: Solid state detector (SSD); Everhart Thornley SE detector (ETD, Thermo Fisher Scientific, Hillsboro, OR, USA); image size: 2560 × 1920 pixel. Morphological pictures were acquired by scattered electron signal (SE signal) while crystal observation was performed by backscattered electrons technique (BSE signal). The WDS analytical conditions (Spectrometers WDS XCE type and X type) were: An accelerating voltage of 15 keV and a Faraday cup current of 10 nA, 11 mm as working distance. All WDS analysis were carried out at room temperature (22 ± 1 °C) and for each sample, three points were analyzed to determine the average value for N, C, and O content, which should be the major elements for isolated glucans.

### 2.8. Cell Culture

Human immortalized keratinocyte cell line HaCat was cultured into 24-well plate (Falcon, Becton-Dickinson, Lincoln Park, NJ, USA) at a concentration of 1 × 10^5^ cells 37 °C, 5% CO_2_, in Dulbecco’s modified Eagle’s (DMEM) supplemented with 10% fetal bovine serum (FBS) and 1% antibiotics (10.000 μg mL^−1^ streptomycin and 10.000 unit mL^−1^ penicillin), and incubated overnight at 37 °C in a humidified 5% CO_2_ atmosphere. Cell counting was achieved using Countess Automated Cell Counter (Thermo Fisher Scientific, Waltham, MA, USA) by Trypan Blue staining. Glucan samples, prepared using water as solvent, were solubilized in complete medium (0.5 mL). 

### 2.9. Cell Viability MTT Assay

Cell viability was estimated by MTT, which is reduced to an insoluble, colored, formazan product by mitochondrial succinate dehydrogenase [26,27,28]. The amount of color produced is directly proportional to the number of viable cells. A day before the assay, HaCaT cells were seeded in 24-well plates at an optimum per 100 μl medium per well. The cell suspensions were then incubated at 37 °C to enable cell attachment. After 24 h, the cells were treated with 100 μl of each concentration of diluted glucan samples. The concentrations considered for crude and soluble glucans from J1, J2, and J3, were 50 µg mL^−1^ and 100 µg mL^−1^. All concentrations were in triplicates. HaCat cells were incubated with prepared samples in 24-well-plates for 24 h. After incubation, the medium was discarded and refreshed with additional 0.5 mL of medium and 50 µL of MTT (5 mg mL^−1^) was added to the wells and incubated for 6 h at 37 °C. After that, the medium was discarded, the cells washed twice using PBS, and 200 µL of DMSO was added to dissolve the colored formazan crystals agitating the plates on a shaker for 5 min, under basic condition [27]. The optical density (OD) was read at 545 nm using a microtiter plate reader (Synergy H1 by BioTeck, Winooski, VT, USA). The results obtained were normalized to percent of control (wells without glucans) and plotted against glucan sample concentration. Cell viability was calculated following the Equation (1):

Viability (%) = (Sample OD/Control OD) × 100(1)

### 2.10. In Vitro Scratch Test

HaCaT cells were plated into three 6-well plate (one for each soluble glucan) in the presence of DMEM, 10% FBS, and incubated at 37 °C in a humidified 5% CO_2_ atmosphere. After attachment of the cells to the plate, DMEM was removed and the adherent cell layer was scraped in a straight line using a sterile 2 μL pipette tip in order to create a scratch. Cellular debris were removed by extensive washing with PBS. Then, the cells were treated with DMEM and 10% FBS containing glucan samples at different concentrations (10, 20, 50, and 100 μg mL^−1^). The cells were then incubated at 37 °C in a humidified 5% CO_2_ atmosphere for 24 and 48 h. The scratch area was periodically imaged, at 0, 24, and 48 h acquired at 40× of magnification (Leica DMIL, Buccinasco (MI), Italy) in clear field. The experiments were performed in triplicate.

### 2.11. Statistical Analysis

Unless otherwise indicated, statistical significance was determined by a two-tailed ANOVA with Bonferroni comparison correction. *p*-value of < 0.05 was regarded as significant. Results are expressed as mean ± SD. 

## 3. Results and Discussion

### 3.1. Extraction of Glucans 

After removal of lipids and soluble compounds with acetone, methanol, and aqueous ethanol, in order to facilitate the complete separation of glucans, the extraction process involved alkaline extraction to remove starch, acidic precipitation of free proteins, and precipitation from absolute ethanol. The raw fractions produced underwent the dialysis process, resulting in soluble fractions, which dissolved in water rapidly and easily. The yields (*w*/*w*) of crude and soluble glucans are reported in Table 2. Data reported in the table highlighted that the glucan content increased as the maturation proceeded. The recovery of crude samples was 13.8 ± 2.9 mg g^−1^ at the first harvesting period, 28.2 ± 1.2 mg g^-1^ at the second harvesting period, and 38.2 ± 0.8 mg g^−1^ at the third. The J1 soluble fraction was found to be one-third of the crude sample (4.7 ± 0.6 mg g^−1^), but J2 and J3 soluble fractions were reduced by only 1.6-fold compared with raw samples (17.8 ± 0.8 mg g^−1^ and 24.4 ± 0.4 mg g^−1^, respectively).

The recovery rate is defined as the ratio between the amount of glucan standard and glucan recovered by extraction from the spiked sample. The average recovery rate was calculated for each stage of harvesting at all validation levels: The value for glucans from J1 was 90 ± 5%, for J2 was 88 ± 4%, and for J3 was 91 ± 6%. The detailed results are reported in Table 3. The data obtained proved the applicability and reproducibility of the extraction procedure used.

### 3.2. FT-IR Spectroscopic Analysis

**Figure 3:**
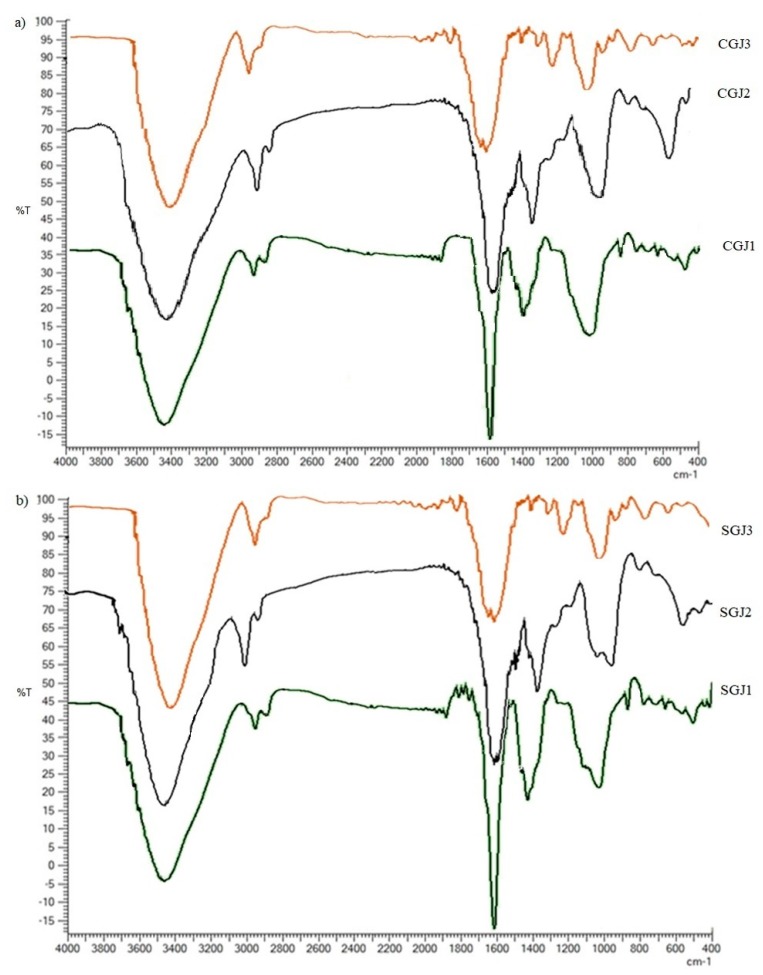
The Fourier transform infrared spectra (FT-IR) of crude (**a**) and soluble (**b**) glucans. CGJ1 (crude glucans from jujubes of first harvesting); CGJ2 (crude glucans from jujubes of second harvesting); CGJ3 (crude glucans of jujubes at third harvesting); SGJ1 (soluble glucans from jujubes of first harvesting); SGJ2 (soluble glucans from jujubes of second harvesting); SGJ3 (soluble glucans from jujubes of third harvesting).

Infrared spectra of crude and soluble glucans showed the characteristic bands of the major functional groups in the 4000–400 cm^−1^ IR region (Figure 3). The IR band centered around 3422–3445 cm^−1^ indicated the symmetrical and asymmetric stretching of the OH groups, contained in a significant number in the glucan backbone. The bands at 2922–2924 cm^−1^ and 2859 cm^−1^ corresponded to CH_2_ stretching vibrations of CH_2_OH groups. The 1500-1800 cm^-1^ region of IR spectra showed two bands at 1609–1640 cm^−1^ (the amide I) and 1509–1527 cm^−1^ (the amide II), which indicated the presence of the amide bond and therefore of proteins in the sample generating the stretching of the CN and NH groups [29,30]. The IR peaks in the region of 1200–1440 cm^−1^ were originated by in-plane ring deformation including CH and OH bending modes. The band around 1034 cm^−1^ in the region of 990–1200 cm^−1^ indicated the COC and CC stretching vibrations of the glucosides ring, which are characteristic for polysaccharides and can be used for their identification [31]. The 950–750 cm^−1^ region, called “anomeric region”, is sensitive to anomeric structure around glycosidic bond of the glucopiranose rings: The α-linkage is indicated by a band around 850–780 cm^−1^ and at 920–870 cm^−1^ for β-linkage. The presence of absorption bands at 895–876 cm^−1^ and at 783–800 cm^−1^ confirmed, respectively, β- and α-configurations of the isolated glucans [32,33]. FT-IR spectra of raw and soluble glucans at all stages of harvesting showed no significant differences regarding the bands characterizing the structure of glucans. Only variations in the absorption intensity of the bands could be observed depending on the maturation stages. Spectroscopic analysis shows that the different collection stages did not involve structural changes.

### 3.3. Detection of Glucan Content

**Figure 4:**
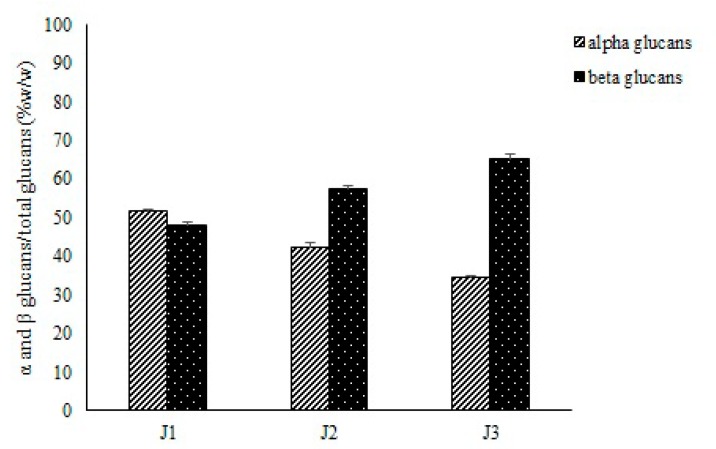
Percentage content of α- and β-glucans compared to total glucans.

A specific enzymatic procedure using yeast glucan assay kit (Megazyme, Astori Tecnica Snc, Poncarale (BS), Italy) was performed in order to determine total glucans and α- and β-glucans. In this approach, controlled acid hydrolysis followed by incubation with specific enzyme ensured complete hydrolysis of glucans to glucose, which was specifically measured with GOPOD reagent. This acid-enzymatic method minimizes the loss of glucose through secondary reactions. It confirmed the results obtained with the alkaline extraction, namely that the total content of the glucans increased as the fruit ripening proceeds (Table 4). The highest glucan content, recovered from J3, was 26.6 mg g^−1^, while the lowest amount was obtained from J1 (19.5 mg g^−1^). Also, the content of α-glucans remained almost constant from the first to the third harvesting stages but compared to total glucans, decreased by about 15%. β-glucan content at the third stage was almost twice as much in relation to the first stage, ranging from 9.4 mg g^−1^ to 17.4 mg g^−1^. β-glucan percentage content with respect to total glucans increased from 48.2 to 65.4% (Figure 4).

### 3.4. Scanning Electron Microscopy

**Figure 5:**
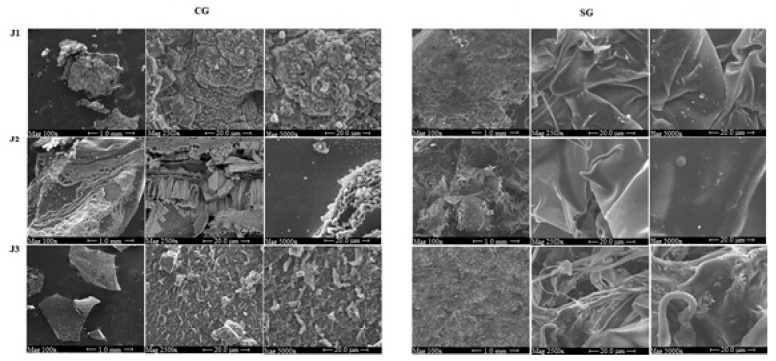
Scanning electron microscopy of crude (CG) and soluble glucans (SG) from J1 (jujubes of first harvesting), J2 (jujube of the second harvesting); J3 (jujube of the third harvesting).

Scanning electron microscopy was performed observing the surface structural differences of the jujube glucans at each stage of harvesting, before and after dialysis. The study conditions were the same for all sample: Three magnifications equal to 100×, 2500×, and 5000× for a scale equal to 1 mm for the first magnification only and 20 μm for the other (Figure 5). The surface of crude glucans (CG) from J1 (CGJ1) showed a granular appearance with an irregular and smaller particle size distribution. In fact, at high magnifications (2500×–5000×), the presence of crystals with the size of the order of microns was evident. The CGJ2 had an amorphous matrix, while maintaining a certain degree of crystallinity. The presence of conchoidal fractures, typically of an amorphous material, characterized the extracted sample from CGJ3 (Figure 5). The higher magnifications showed areas with microcrystalline structure. The structural differences between the three glucans, ranging from “pseudo-crystalline” in CGJ1, to the predominantly amorphous of CGJ3, through an intermediate structure, both amorphous and crystalline, of CGJ2, were probably a consequence of the different stage of harvesting that affect the microstructures of glucans. Soluble glucans (SG) from J1, J2, and J3 (SGJ1, SGJ2, and SGJ3, respectively) were quite similar at low magnifications (100X). The differences could be observed just at higher magnifications (2500×–5000×). The surface of SGJ1 showed a sheeting structures, while the one of SGJ2 evidenced changes in the morphology characterized by a film structure on which some filaments were visible. The filamentous structures appeared dominant on the surface of samples of SGJ3. Anyhow, the morphological differences between crude and soluble samples were evident (Figure 5). 

The variation of the elemental composition in nitrogen, carbon, and oxygen in the glucan matrix before and after the harvesting as a function of the harvesting period was evaluated by wavelength dispersive spectroscopy (WDS). Table 5 shows the results of WDS point analyses, which were an average of five measurements in different points of each sample. The results showed that the main element in all samples, both crude and soluble glucans, was the carbon. Its percentage ranged from 17.9 ± 0.4 in CGJ1 to 19.3 ± 1 in CGJ2, while it increased in soluble samples. The oxygen percentage remained almost constant in the 10.4%–14.4% range in all samples, both crude and soluble. Nitrogen represented a small percentage compared to carbon and oxygen content, ranging from 0.4% in CGJ3 and SGJ3 to 1.5% in CGJ2. The results highlighted that harvesting stage determined some changes in elemental composition of glucans.

### 3.5. Cell Viability

**Figure 6:**
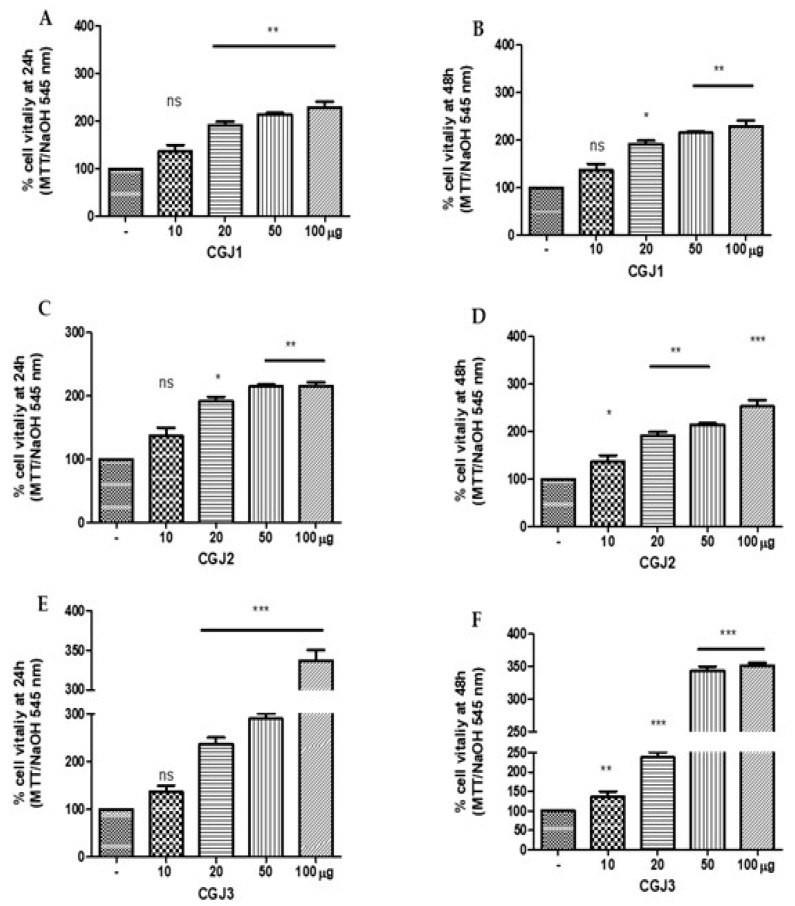
MTT assay of HaCat cells line treated with crude glucans (**A**) CGJ1 (crude glucans from jujubes of first harvesting); (**C**) CGJ2 (crude glucans from jujubes of second harvesting); (**E**) CGJ3 (crude glucans of jujubes at third harvesting) after 24 h and (**B**) CGJ1, (**D**) CGJ2, and (**F**) CGJ3 after 48 h. * *p* < 0.05, ** *p* < 0.01 and *** *p* < 0.001 in comparison with control.

**Figure 7:**
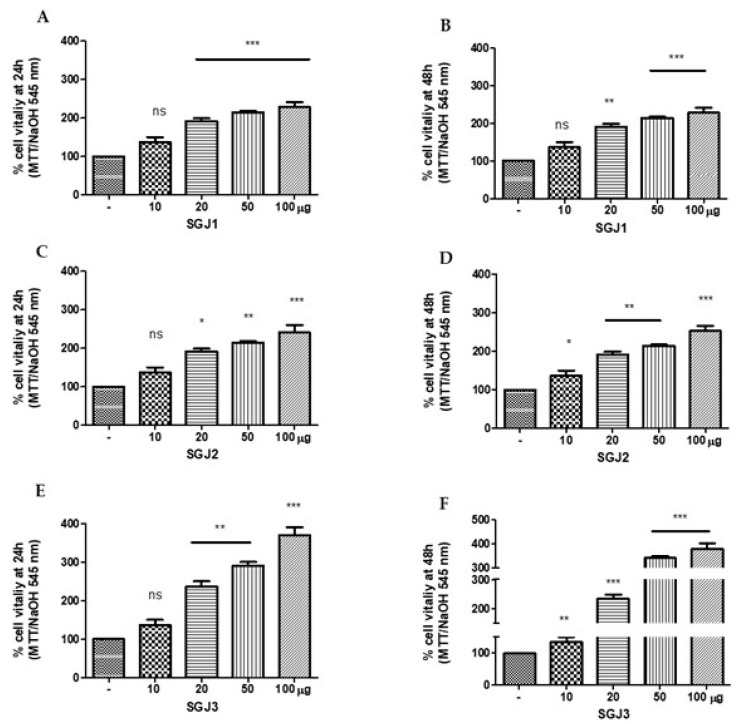
MTT assay of HaCat cells line treated with crude glucans (**A**) from SGJ1 (soluble glucans from jujubes of first harvesting); (**C**) SGJ2 (soluble glucans from jujubes of second harvesting); (**E**) SGJ3 (soluble glucans from jujubes of third harvesting) after 24 h and (**B**) SGJ1, (**D**) SGJ2, and (**F**) SGJ3 after 48 h. * *p* < 0.05, ** *p* < 0.01 and *** *p* < 0.001 in comparison with control.

Keratinocytes constitute the major cellular component in the epidermis and are involved in the process of wound healing by providing a continuous supply of proliferative cells. For this reason, HaCat cell line were used as a model for studying the regenerative potential of glucans extracted from Italian jujube fruits at three harvesting periods and purified by dialysis process. HaCat cells were treated with different amount of CGJ1, CGJ2, and CGJ3 (10, 20, 50, and 100 µg). After 24 h, any difference was observed at 10 µg for CGJ1, CGJ2, and CGJ3, (Figure 6A,C,E), but a significant increase of cell viability was found at 20, 50, and 100 µg with different statistic values (** *p* < 0.01 at 20, 50, 100 µg of CGJ1; * *p* < 0.05 at 20 µg; and ** *p* < 0.01 at 50 and 100 µg of CGJ2; *** *p* < 0.001 at 20, 50, and 100 µg of CGJ3) (Figure 6A,C,E). The findings demonstrated that the activity of CGJ1, CGJ2, and CGJ3, at 24 h, is dose dependent. After 48 h, proliferative effect was observed even at 10 µg of CGJ2 and CGJ3 (* *p* < 0.05 and ** *p* < 0.01, respectively); however, the findings were significantly highest at 100 µg of CGJ2 and CGJ3 (*** *p* < 0.001, Figure 6D,F). On the contrary, after 48 h of treatment with CGJ3, showed a dose-independent effect, as shown in Figure 6F. In any case, the most significant stimulus to cell viability came from CGJ3 both after 24 and 48 h (336.7% and 351.7%, respectively). In agreement with a previous report showing that β-glucans promote cell proliferation in different cell types [34], this biochemical behavior could be due to the higher number of β-glucans in CGJ3 fraction (Table 4) if compared to CGJ1 and CGJ2. Similar proliferative effects were observed for HaCaT cells treated with the corresponding soluble glucans SGJ1, SGJ2, and SGJ3 (Figure 7). The findings were not statistically significant at 10 µg of SGJ1, SGJ2, and SGJ3 after 24h (Figure 7A,C,E), but cells treated with higher doses of soluble glucans showed a significant increase of cell viability with different statistic values (*** *p* < 0.001 at 20, 50, 100 µg of SGJ1; * *p* < 0.05 at 20 µg, ** *p* < 0.01 at 50; and *** *p* < 0.001 at 100 µg of SGJ2, respectively; ** *p* < 0.01 at 20, 50 µg and *** *p* < 0.001 at 100 µg of SGJ3). The best proliferative effects have been achieved by treating HaCaT cells with 100 µg of SGJ3 after 24 and 48 h (370% and 379.7%, respectively). The results showed that all the glucans displayed no toxicity for HaCaT cells using an MTT assay.

### 3.6. Scratch Assay

**Figure 8:**
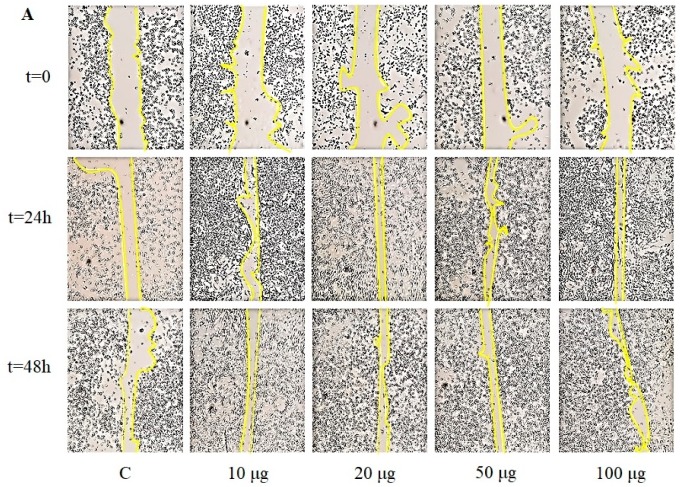

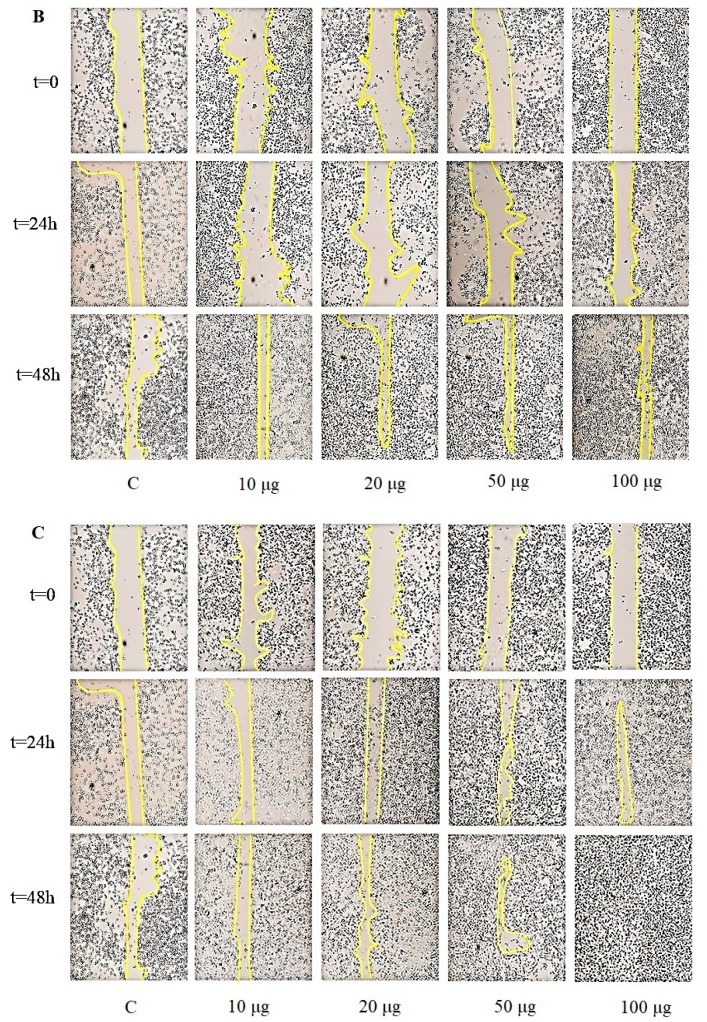
Representative images of time course scratch assay of HaCat cells treated with different concentration of soluble glucans (**A**) SGJ1 (soluble glucans from jujubes of first harvesting); (**B**) SGJ2 (soluble glucans from jujubes of second harvesting); and (**C**) SGJ3 (soluble glucans from jujubes of third harvesting), compared to control (C = untreated). Yellow line indicates the site of the scratch wound.

The scratch wound healing assay has been used to evaluate the migration of cells, and to establish the influence of the traditional medicines on the keratinocytes. Owing to a slightly better proliferative effect on HaCaT cells of the soluble glucans (above all SGJ3) than the crude fractions, scratch assay was conducted on SG fraction-treated keratinocytes, in order to confirm that this fraction was involved in skin regeneration. The HaCat culture monolayer was incised in the central area in the presence of different amount of SGJ1, SGJ2, and SGJ3 (10, 20, 50, and 100 µg). The results are shown in Figure 8A–C. The scratch area was filled with migrating HaCaT cells. 

The images show that the cells treated with SGJ1 and SGJ2 exhibited visible closure of the scratch area compared to control already after 24 h, but the best result was recorded at 48 h in which cellular migration was slowed down but kept stable (Figure 8A,B). Among all the groups, the cells treated with SGJ3 exhibited an excellent response already after 24 h at 100 µg. The wound area was completely restored after 48 h in the cells treated with 50 and 100 µg of SGJ3 (Figure 8C). Therefore, the present study showed that soluble glucans extracted from jujubes prompt cellular survival and cell migration, which are critical in wound healing process. Cell migration is a hallmark of wound repair, which is a complex cellular and biochemical process necessary to restore structurally damaged tissue. What we noticed was a different capability of HaCaT cell migration: The properties of keratinocytes in chronic wounds can depend on the context in which the cell line is migrating because the cell migration occurs in different morphological variants [35]. A successful wound closure needs a crosstalk between the keratinocytes and other cell types involved in wound healing. Thus, the different results obtained for the groups treated with SG from J1, J2, and J3 were probably due to their diversified morphological structure (Figure 5), observing that SGJ3, which is the most effective in improving the wound healing abilities of keratinocytes, is also richer in layers. This structural aspect could influence the cell migration. Glucans, particular β-glucans in which the J3 fraction is rich, are able to act into the maturation of monocyte-derivatives dendritic cells as well as macrophage chemotactic recall into skin damage, both wound and surgical cut [36,37]. Furthermore, it is worth noting that only recently, four types of β-glucans extracted from different cereals promote epithelial cell migration. Moreover, Seo et al. did not record, in their set of experiments, the capability of HaCat to improve cell viability as it is evident in our results [18]. 

## 4. Conclusions

The results of the present study have conclusively indicated that the *Ziziphus jujuba* fruits could serve as a good source for the extraction of nutraceuticals and pharmaceuticals, such as glucans. It was found that their total content increased as the maturation proceeded, as well as the percentage of β- compared to α- fraction. According to the anomeric structure of glucose units, FT-IR spectra of extracted glucans highlighted the simultaneous presence of both the α- and β-glycosidic anomeric bonds in all stages of harvesting, as confirmed by enzymatic assay. SEM showed significant morphological differences between the crude and the soluble glucans at each stage of maturation: The crude samples ranged from "pseudo-crystalline" in CGJ1, to amorphous structure of CGJ3, while soluble glucans exhibited a sheeting structure that becomes filamentous in SGJ3. The efficiency of different concentrations of crude and soluble glucans on survival and proliferation of the keratinocytes (HaCaT cells) was determined by MTT assay. The findings demonstrated that all fractions display no toxicity for the cells, but the best proliferative effects have been achieved by treating keratinocytes with higher doses of SGJ3 both after 24 and 48 h. Among all the groups, the cells treated with the fraction containing the highest percentage of β-glucans (SGJ3), exhibited a good response promoting the complete closure of the wound area after 48 h. Therefore, the present study showed that soluble glucans extracted from jujubes prompt cellular survival and cell migration, which are critical in wound healing process. These findings suggest that glucans from *Ziziphus jujuba* fruits was a potential therapeutic agent for skin repair. 

## Figures and Tables

**Table 1:** Length and diameter of jujube fruits (*Ziziphus jujuba* Mill.) during ripening.

Harvesting	J1	J2	J3
Length * (mm)	19 ± 1	19.7 ± 0.4	21.2 ± 0.7
Diameter * (mm)	4.7 ± 0.5	6.5 ± 0.2	7.8 ± 0.2

* average of *n* measurement ± SD. *n* = 20.

**Table 2:** Recovery of crude and soluble glucans from jujube fruits at three harvesting periods.

Harvesting period	Glucans	Recovery ^a^ (mg g^−1^)	Yield (%)
**J1**	*Crude*	13.8 ± 2.9	1.4
	*Soluble*	4.7 ± 0.6	0.5
**J2**	*Crude*	28.2 ± 1.2	2.8
	*Soluble*	17.8 ± 0.8	1.8
**J3**	*Crude*	38.2 ± 0.8	3.8
	*Soluble*	24.4 ± 0.4	2.4

^a^ Average values of three extractions ± SD.

**Table 3:** Validation results for recovery results ^a^.

	Level (mg g^−1^)											
	30		60		90		120		150		Average	
	R	CV	R	CV	R	CV	R	CV	R	CV	R_tot_	CV
**J1**	87	5	88	3	92	8	90	3	93	6	90	5
**J2**	89	3	86	5	85	3	88	4	92	5	88	4
**J3**	92	5	88	7	89	4	95	8	91	6	91	6

^a^ R = average of 5 experiments (%); CV = coefficient of variation (%). J1 (jujube from first harvesting); J2 (jujube from the second harvesting); J3 (jujube from the third harvesting).

**Table 4:** α- and β-Glucan content from jujubes.

Samples	Total Glucans ^a^ (mg g^−1^)	α-Glucans ^a^ (mg g^−1^)	β-Glucans ^a^ (mg g^−1^)
**J1**	19.5 ± 2.0	10.1 ± 0.5	9.4 ± 0.8
**J2**	21.2 ± 1.3	9.0 ± 0.9	12.2 ± 0.9
**J3**	26.6 ± 1.6	9.2 ± 0.5	17.4 ± 1.1

^a^ average of 3 experiments ± SD.

**Table 5:** Wavelength dispersive spectroscopy (WDS) measurements of the compositions in *wt%* of CGJ1 (crude glucans from jujubes of first harvesting); CGJ2 (crude glucans from jujubes of second harvesting); CGJ3 (crude glucans of jujubes at third harvesting); SGJ1 (soluble glucans from jujubes of first harvesting); SGJ2 (soluble glucans from jujubes of second harvesting); SGJ3 (soluble glucans from jujubes of third harvesting).

Elements				Samples		
	CGJ1	SGJ1	CGJ2	SGJ2	CGJ3	SGJ3
N	0.7 ± 0.2	0.6 ± 0.1	1.5 ± 0.2	1.1 ± 0.2	0.4 ± 0.1	0.4 ± 0.1
C	17.9 ± 0.4	36.7 ± 3.2	19.3 ± 1	33.4 ± 3	16.7 ± 1.7	23.3 ± 1.3
O	10.4 ± 0.7	14.4 ± 2	10.7 ± 1.5	10.5 ± 1.5	10.4 ± 0.7	11.5 ± 0.9

The results are average of 5 measurements in different points of each sample ± SD.

## Abbreviations

J1	jujube from first harvesting
J2	jujube from the second harvesting
J3	jujube from the third harvesting
MTT	3-(4,5-dimethylthiasol-2-yl)-2,4-diphenyltetrazolium bromide
MWCO	Molecular Weight Cut Off
FT-IR	Fourier Transform Infrared Spectroscopy
SEM	Scanning Electron Microscopy
WDS	wavelength dispersive spectroscopy
GOPOD	glucose-oxidase/peroxidase-reagent
CGJ1	crude glucans from jujubes of first harvesting
CGJ2	crude glucans from jujubes of second harvesting
CGJ3	crude glucans from jujubes of third harvesting
SGJ1	soluble glucans from jujubes of first harvesting
SGJ2	soluble glucans from jujubes of second harvesting
SGJ3	soluble glucans from jujubes of third harvesting
